# Rapid postoperative platelet decline in a lung adenocarcinoma with sarcomatoid differentiation and concomitant JAK2 V617F mutation: a case report

**DOI:** 10.3389/fonc.2025.1722712

**Published:** 2025-12-08

**Authors:** Luojie Deng, Xiaoping Li

**Affiliations:** 1 Department of Thoracic Surgery, Tianjin First Central Hospital, Tianjin, China; 2 Nankai University School of Medicine, Tianjin, China

**Keywords:** lung adenocarcinoma, sarcomatoid carcinoma, TP53, JAK2, Met, reactive thrombocytosis

## Abstract

**Background:**

Lung cancer is one of the leading causes of cancer incidence and mortality worldwide. At the molecular level, its development involves aberrations in multiple oncogenic pathways. Here, we report a rare case of lung adenocarcinoma with sarcomatoid differentiation and profound thrombocytosis (1,350 × 10^9/^L), harboring concurrent TP53, JAK2 V617F, and MET exon 14 skipping mutations.

**Case presentation:**

A 75-year-old female underwent VATS wedge resection for pathological stage IB lung adenocarcinoma. Platelet counts dropped from 1,350 to 572 × 10^9/^L within 3 weeks postoperatively and remained stable during the 6-month follow-up period. No adjuvant therapy was administered. Molecular profiling of the resected tumor revealed concurrent TP53, JAK2, and MET mutations.

**Conclusion:**

This case represents a rare coexistence of JAK2 V617F, MET, and TP53 mutations in lung adenocarcinoma. A marked postoperative decline in platelet count was observed without adjuvant therapy, suggesting a possible tumor-associated reactive thrombocytosis rather than a primary hematologic disorder. Recognition of such platelet dynamics may aid in the clinical assessment and follow-up of lung cancer patients.

## Introduction

Reactive thrombocytosis in solid tumors is typically mediated by IL-6-driven thrombopoietin production. While JAK2 V617F mutation is well-established in myeloproliferative neoplasms (>95% polycythemia vera), its occurrence in non-hematopoietic malignancies is exceptionally rare. However, the biological significance of concurrent JAK2/TP53/MET alterations in NSCLC remains unexplored.

TP53 mutations are among the most frequent genetic alterations in lung cancer and are linked to genomic instability and aggressive tumor behavior (1, 2).The JAK2 p.V617F mutation is a gain-of-function alteration well recognized in myeloproliferative neoplasms (MPNs) but rarely detected in solid tumors. Its presence may cause constitutive activation of JAK-STAT signaling and excessive thrombopoiesis (3, 4). In classical MPNs, the mutation is detected in approximately 70% with essential thrombocythemia (5).Several signaling interactions, including PI3K/AKT/mTOR and MAPK, have been implicated in platelet production and inflammatory regulation, though their relevance in lung cancer remains unclear (6, 7).

Such dysregulated signaling may underlie paraneoplastic hematologic phenomena observed in solid tumors. In particular, thrombocytosis—a peripheral platelet count exceeding 450 × 10^9/^L—is frequently reported in malignancies and is thought to reflect tumor-driven cytokine release, inflammation, and overall tumor burden (8).

Here, we report a case of lung adenocarcinoma with sarcomatoid differentiation and marked thrombocytosis harboring concurrent JAK2 V617F, TP53, and MET exon 14 skipping mutations, aiming to describe its clinical course and explore the potential association between tumor activity and platelet dynamics.

## Case presentation

**Figure 1:**
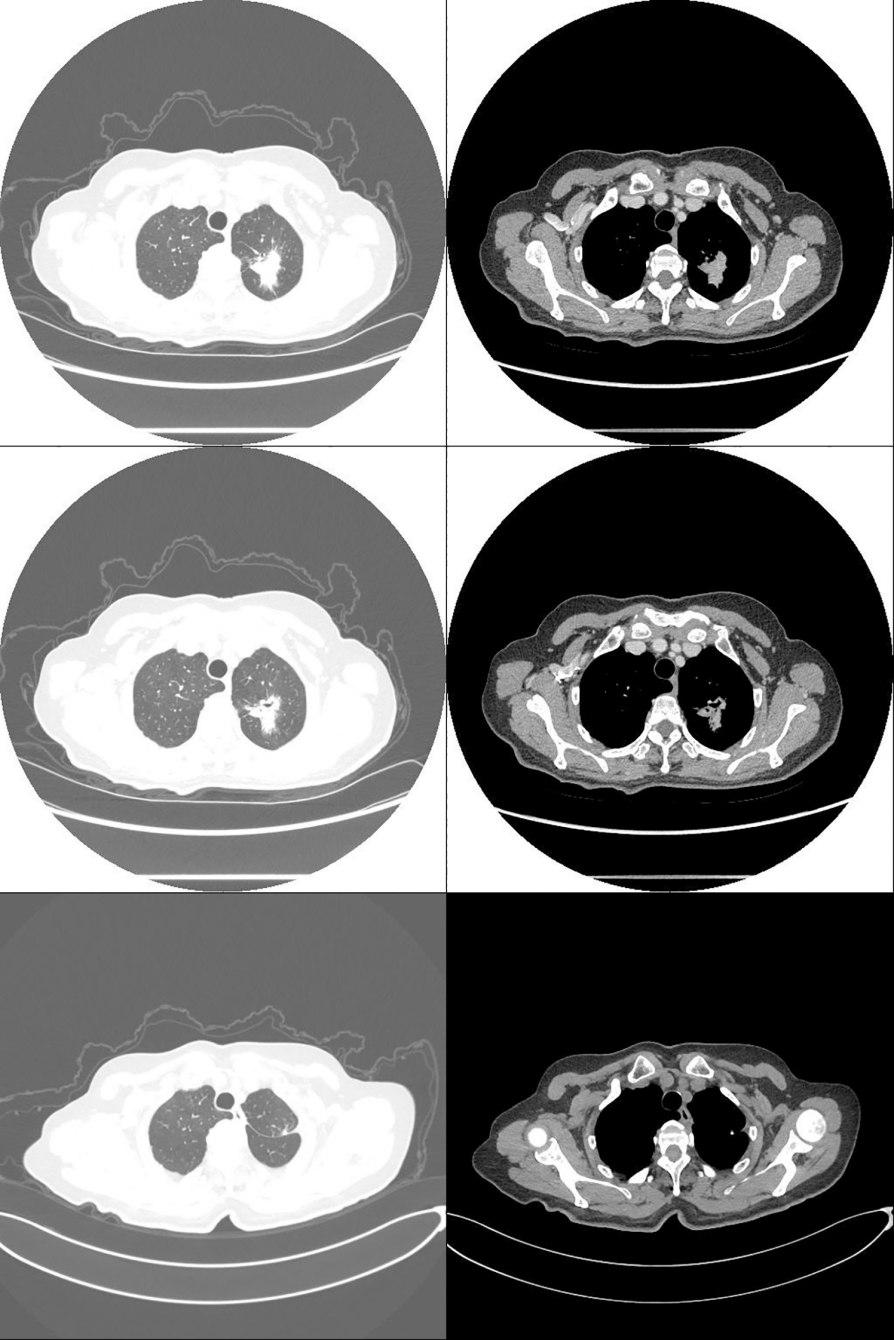
Computed tomography: non-small cell lung cancer (NSCLC) in the left upper lobe (pT2aN0M0, stage IB; 30.1 × 22.9 mm). Preoperative (top two rows) and postoperative (bottom row) images.

A 75-year-old female patient presented with a one-week history of cough and productive sputum with intermittent hemoptysis. Chest computed tomography (CT) demonstrated a patchy soft tissue density mass in the apicoposterior segment of the left upper lobe, measuring approximately 30.1×22.9 mm (**Figure 1**). The lesion exhibited ill-defined margins, spiculation, and partial ground-glass opacity, without evidence of lymphadenopathy or distant metastasis. The patient had no history of smoking, chronic inflammatory disease, autoimmune disorders, or bleeding diathesis.

On admission, physical examination was unremarkable. Laboratory tests revealed marked thrombocytosis, with a platelet count of 1,350 × 10^9/^L (reference range: 120–350 × 10^9/^L) and plateletcrit of 1.044%. Leukocytosis was noted with a total white blood cell count of 15.96 ×10^9/^L and neutrophil count of 12.53 × 10^9/^L, while lymphocytes, red blood cells, and hemoglobin remained within normal ranges. Serum tumor markers were as follows: alpha-fetoprotein (AFP), 8.51 ng/mL; carcinoembryonic antigen (CEA), 5.79 ng/mL; neuron-specific enolase (NSE), 34.40 ng/mL; and pro-gastrin-releasing peptide (ProGRP), 76.50 ng/mL. Common causes of thrombocytosis, including iron deficiency and inflammatory disorders, were excluded based on clinical and laboratory findings. However, no bone marrow biopsy or peripheral smear was performed.

The patient underwent video-assisted thoracoscopic wedge resection of the left upper lobe with systematic lymph node dissection. Intraoperative frozen section analysis revealed invasive non-mucinous adenocarcinoma, predominantly lepidic with a mixed acinar growth pattern. Foci of pleomorphic/spindle cell carcinoma were also identified, consistent with sarcomatoid carcinoma. Focal invasion of the visceral pleura was present. The pathological stage was pT2aN0M0 (stage IB).No adjuvant therapy was administered postoperatively. Postoperative molecular profiling (NGS) identified TP53 c.673-1G>T, JAK2 p.V617F, and MET exon 14 skipping mutations.

**Figure 2:**
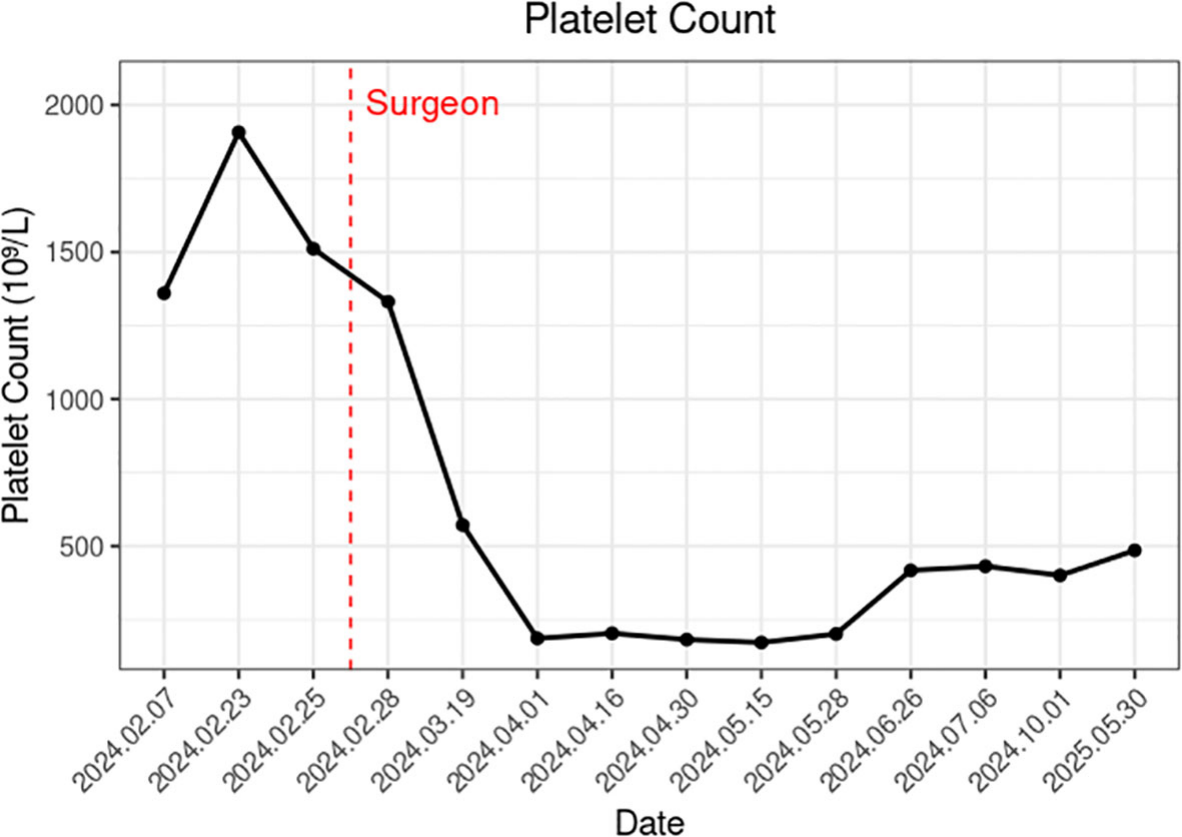
Dynamic changes in platelet counts (the red dashed line indicates the date of surgery on February 24, 2024).

Three weeks after surgery, follow-up laboratory testing showed a reduction in platelet count to 572 × 10^9/^L (**Figure 2**). Chest CT demonstrated interval shrinkage of the left upper lobe consolidation (**Figure 1**), and tumor markers normalized (CEA: 3.31 ng/mL; NSE: 12.40 ng/mL). The patient received hydroxyurea and enteric-coated aspirin for six months postoperatively, after which treatment was discontinued as the platelet count progressively declined and stabilized. No adjuvant chemotherapy or targeted therapy was administered. The patient has since remained under surveillance with no evidence of tumor recurrence.

## Discussion

This report describes a rare case of lung adenocarcinoma with concurrent TP53, MET exon 14 skipping mutations and JAK2 V617F mutations, accompanied by marked preoperative thrombocytosis that decreased following tumor resection.

The TP53 c.673-1G>T splice-site alteration likely disrupted normal splicing and contributed to tumor progression (2). The marked preoperative thrombocytosis observed in this patient was most likely secondary to tumor-related inflammatory activation, potentially amplified by JAK2 signaling. The presence of a MET exon 14 skipping mutation—a known oncogenic driver frequently detected in pulmonary sarcomatoid carcinoma (PSC)—further supports an aggressive histologic subtype (9, 10). The postoperative decline in platelet count is consistent with a reactive process rather than a primary hematologic disorder. Dynamic platelet fluctuations in this context may serve as a clinical indicator—or “mirror”—of tumor activity, provided that other reactive causes such as iron deficiency, infection, or autoimmune disorders are excluded. This observation may also be considered in the context of tumor-educated platelets (TEPs), which have been associated with cancer-related thrombosis and inflammatory activation (11).

Given this mutational profile and the dynamic hematologic changes, several plausible mechanisms may be proposed to explain the observed thrombocytosis.

Among these, one plausible mechanism involves cytokine-mediated activation of the JAK/STAT pathway through interleukin-6 (IL-6) signaling. Notably, both TP53 and MET mutations have been reported to upregulate interleukin-6 (IL-6) expression (12, 13). IL-6, as a key upstream regulator of JAK/STAT signaling, can activate STAT3 and related downstream molecules, enhance thrombopoietin expression, and drive excessive myeloid proliferation, ultimately leading to secondary (reactive) thrombocytosis (14).

Beyond cytokine signaling, intracellular oncogenic pathways may also play an important role in promoting excessive megakaryopoiesis. Mutations in TP53, MET and JAK2 may aberrantly activate the PI3K/AKT/mTOR signaling cascade, thereby driving megakaryocytic expansion and thrombocytosis. TP53 mutations impair the tumor suppressor function of p53, weakening its negative regulatory influence on the PI3K/AKT/mTOR axis, for example through PTEN downregulation, which facilitates unchecked mTOR activation and enhances megakaryocyte proliferation and survival (15, 16). MET exon 14 skipping mutations stabilize the c-MET receptor by preventing its degradation, resulting in sustained activation of the PI3K/AKT/mTOR pathway and accelerated hematopoietic progenitor proliferation with megakaryocytic differentiation (10). The JAK2 V617F mutation exerts multifaceted effects: it suppresses GADD45g, releasing its inhibitory interaction with RAC2 and thereby activating PAK1–PI3K/AKT signaling (17); in addition, constitutive STAT5 activation promotes DDX5 (p68) expression, which maintains mTOR activity and amplifies cellular responsiveness to growth signals (18). Collectively, these mechanisms converge to sustain aberrant PI3K/AKT/mTOR activation, leading to excessive megakaryopoiesis and pathological thrombocytosis in patients carrying such mutations.

It is conceivable that the TP53 splice-site and MET exon 14 skipping mutations primarily contributed to tumorigenesis and cytokine dysregulation, whereas the JAK2 V617F mutation amplified inflammatory and thrombopoietic signaling. These combined alterations may have created a self-reinforcing loop in which tumor-derived cytokines activated the JAK/STAT pathway, while concurrent activation of PI3K/AKT/mTOR signaling promoted megakaryocytic proliferation and platelet production.

These findings suggest—but do not confirm—a possible interplay between tumor-associated oncogenic signaling and hematopoietic activation. The precise molecular mechanisms remain speculative in the absence of IL-6 quantification or phosphorylated STAT3 analysis, and further functional studies are warranted to validate these hypotheses.

Limitations of this report include the lack of hematologic confirmation by bone marrow biopsy and absence of cytokine measurements. Thus, the observed association should be interpreted as descriptive rather than causal. Despite these limitations, the postoperative drop in platelet count provides indirect clinical evidence supporting a reactive process secondary to tumor removal.

**Figure 3:**
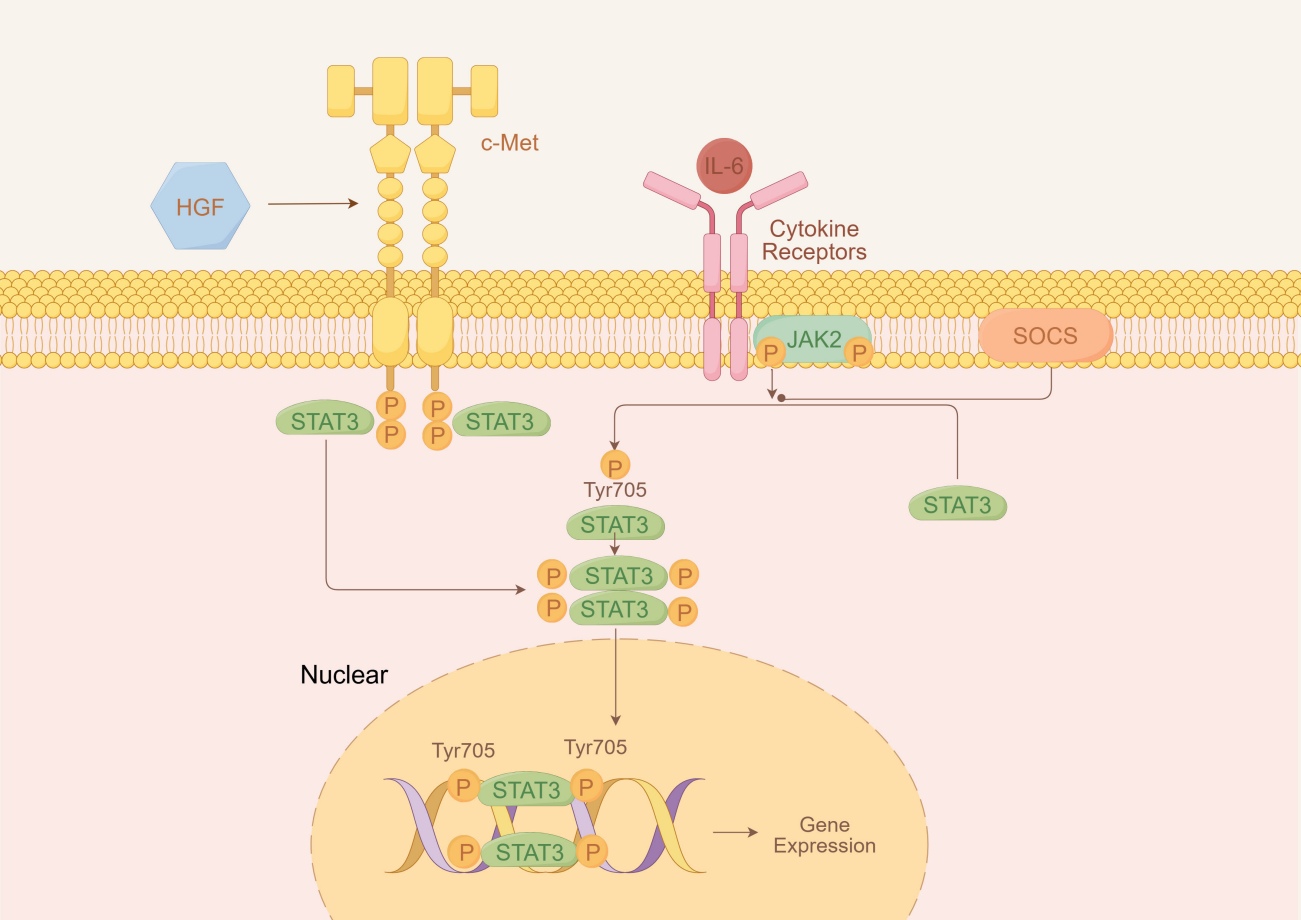
Hypothetical schematic of IL-6/JAK/STAT3 signaling pathway.

**Figure 4:**
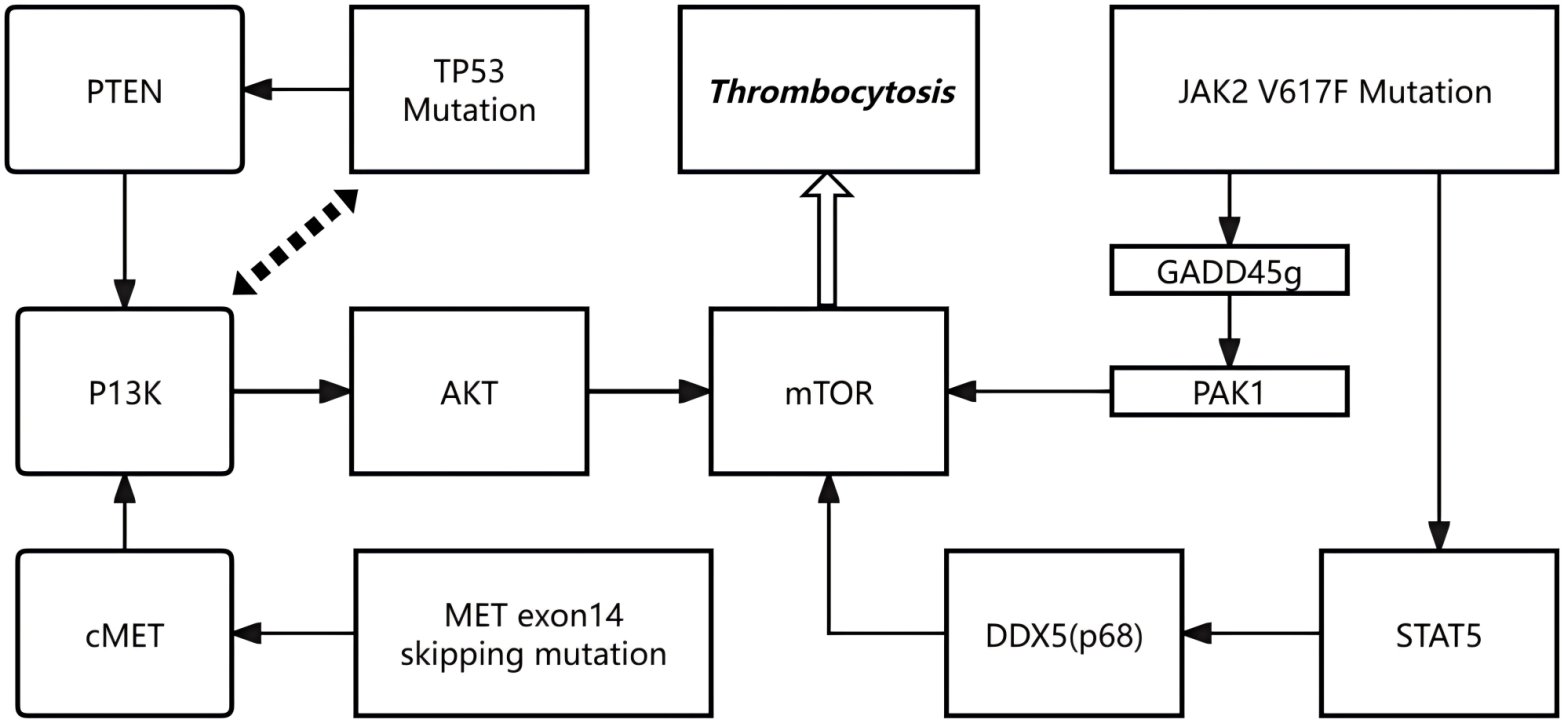
Hypothetical schematic of PI3K/AKT/mTOR signaling pathway.

**Figures 3**, **4** are retained to conceptually illustrate potential signaling interactions; these hypotheses require further experimental validation.

## Conclusion

This case illustrates a rare coexistence of JAK2 V617F, TP53, and MET exon 14 skipping mutations in NSCLC, associated with marked preoperative thrombocytosis that declined after surgery. Although a tumor-associated mechanism is plausible, further studies are needed to confirm this relationship and to determine whether platelet trends may serve as indicators of disease activity.

## Data Availability

The original contributions presented in the study are included in the article/supplementary material. Further inquiries can be directed to the corresponding author.

## References

[1] Wang R Pan S Song X . Research advances of EGFR-TP53 co-mutation in advanced non-small cell lung cancer. Zhongguo Fei Ai Za Zhi. (2022) 25:174–82. doi: 10.3779/j.issn.1009-3419.2022.101.06, PMID: 35340160 PMC8976205

[2] Soussi T Wiman KG . TP53: an oncogene in disguise. Cell Death Differ. (2015) 22:1239–49. doi: 10.1038/cdd.2015.53, PMID: 26024390 PMC4495363

[3] Kralovics R Passamonti F Buser AS Teo SS Tiedt R Passweg JR . A gain-of-function mutation of JAK2 in myeloproliferative disorders. N Engl J Med. (2005) 352:1779–90. doi: 10.1056/NEJMoa051113, PMID: 15858187

[4] Thomas SJ Snowden JA Zeidler MP Danson SJ . The role of JAK/STAT signalling in the pathogenesis, prognosis and treatment of solid tumours. Br J Cancer. (2015) 113:365–71. doi: 10.1038/bjc.2015.233, PMID: 26151455 PMC4522639

[5] Greenfield G McMullin MF Mills K . Molecular pathogenesis of the myeloproliferative neoplasms. J Hematol Oncol. (2021) 14:103. doi: 10.1186/s13045-021-01116-z, PMID: 34193229 PMC8246678

[6] Zhang Y Zhao Y Liu Y Zhang M Zhang J . New advances in the role of JAK2 V617F mutation in myeloproliferative neoplasms. Cancer. (2024) 130:4229–40. doi: 10.1002/cncr.35559, PMID: 39277798

[7] Blaquier JB Recondo G . Non-small-cell lung cancer: how to manage MET exon 14 skipping mutant disease. Drugs Context. (2022) 11:2022–2–2. doi: 10.7573/dic.2022-2-2, PMID: 35855460 PMC9255265

[8] Li SD Ma M Li H Waluszko A Sidorenko T SChadt EE . Cancer gene profiling in non-small cell lung cancers reveals activating mutations in JAK2 and JAK3 with therapeutic implications. Genome Med. (2017) 9:89. doi: 10.1186/s13073-017-0478-1, PMID: 29082853 PMC5662094

[9] Gong C Xiong H Qin K Wang J Cheng Y Zhao J . MET alterations in advanced pulmonary sarcomatoid carcinoma. Front Oncol. (2022) 12:1017026. doi: 10.3389/fonc.2022.1017026, PMID: 36212500 PMC9539670

[10] Organ SL Tsao MS . An overview of the c-MET signaling pathway. Ther Adv Med Oncol. (2011) 3:S7–S19. doi: 10.1177/1758834011422556, PMID: 22128289 PMC3225017

[11] Najafi S Asemani Y Majidpoor J Mahmoudi R Aghaei-Zarch SM Mortezaee K . Tumor-educated platelets. Clin Chim Acta. (2024) 552:117690. doi: 10.1016/j.cca.2023.117690, PMID: 38056548

[12] Huber A Allam AH Dijkstra C Thiem S Huynh J Poh AR . Mutant TP53 switches therapeutic vulnerability during gastric cancer progression within interleukin-6 family cytokines. Cell Rep. (2024) 43:114616. doi: 10.1016/j.celrep.2024.114616, PMID: 39128004 PMC11372443

[13] Hov H Tian E Holien T Holt RU Våtsveen TK Fagerli UM . c-Met signaling promotes IL-6-induced myeloma cell proliferation. Eur J Haematol. (2009) 82:277–87. doi: 10.1111/j.1600-0609.2009.01212.x, PMID: 19187270 PMC2704927

[14] Johnson DE O’Keefe RA Grandis JR . Targeting the IL-6/JAK/STAT3 signalling axis in cancer. Nat Rev Clin Oncol. (2018) 15:234–48. doi: 10.1038/nrclinonc.2018.8, PMID: 29405201 PMC5858971

[15] Stambolic V MacPherson D Sas D Lin Y Snow B Jang Y . Regulation of PTEN transcription by p53. Mol Cell. (2001) 8:317–25. doi: 10.1016/s1097-2765(01)00323-9, PMID: 11545734

[16] He Y Sun MM Zhang GG Yang J Chen KS Xu WW . Targeting PI3K/Akt signal transduction for cancer therapy. Signal Transduct Target Ther. (2021) 6:425. doi: 10.1038/s41392-021-00828-5, PMID: 34916492 PMC8677728

[17] Zhang P You N Ding Y Zhu W Wang N Xie Y . Gadd45g insufficiency drives the pathogenesis of myeloproliferative neoplasms. Nat Commun. (2024) 15:2989. doi: 10.1038/s41467-024-47297-2, PMID: 38582902 PMC10998908

[18] Takeda K Tago K Funakoshi-Tago M . The indispensable role of the RNA helicase DDX5 in tumorigenesis induced by the myeloproliferative neoplasm-associated JAK2V617F mutant. Cell Signal. (2023) 102:110537. doi: 10.1016/j.cellsig.2022.110537, PMID: 36442590

